# A Case Series Report on the Effect of Tofacitinib on Joint Inflammation and Gut Microbiota Composition in Psoriatic Arthritis Patients Naive to Biologic Agents

**DOI:** 10.3390/microorganisms12122387

**Published:** 2024-11-21

**Authors:** Andrea Picchianti Diamanti, Concetta Panebianco, Valeria Di Gioia, Ilaria Anna Bellofatto, Simonetta Salemi, Roberta Di Rosa, Giorgio Sesti, Gabriele Nalli, Gerardo Salerno, Etta Finocchiaro, Bruno Laganà

**Affiliations:** 1 Department of Clinical and Molecular Medicine, S. Andrea University Hospital, “Sapienza” University of Rome, 00189 Rome, Italy; valeria.digioia@hotmail.it (V.D.G.); simonettasalemi@gmail.com (S.S.); roberta.dirosa@uniroma1.it (R.D.R.); giorgio.sesti@uniroma1.it (G.S.); gabriele.nalli@uniroma1.it (G.N.); gerardo.salerno@uniroma1.it (G.S.); bruno.lagana@uniroma1.it (B.L.); 2 Division of Gastroenterology, Fondazione IRCCS Casa Sollievo della Sofferenza Hospital, 71013 San Giovanni Rotondo, Italy; panebianco.c@gmail.com; 3 Department of Internal Medicine, University of Genoa, 6 Viale Benedetto XV, 16132 Genoa, Italy; ilaria.bellofatto@live.it; 4 Dietetic and Clinical Nutrition Unit, City of Health and Science University Hospital, 10126 Turin, Italy; ettafinocchiaro@gmail.com

**Keywords:** gut microbiota, psoriatic arthritis, musculoskeletal ultrasonography, JAK inhibitors, tofacitinib

## Abstract

Introduction: Psoriatic arthritis (PsA) is a complex condition within the Spondyloarthritis (SpA) group. Recent studies have focused on the important role of the intestinal microbiota in maintaining immunological homeostasis, highlighting how intestinal dysbiosis may act as a trigger for autoimmune diseases. Tofacitinib is a Janus kinase inhibitor (JAK-i) with proven efficacy for the treatment of both rheumatoid arthritis and PsA. However, there is a lack of data on its ability to reduce joint remission through ultrasonography (US) and the effects it might have on the composition of the gut microbiota. Methods: Here, we present a case series of seven bio-naïve PsA patients who received tofacitinib treatment and were followed up for 12 months. The clinical response was assessed using validated scores (DAPSA, ASDAS, and BASDAI), laboratory tests, and US assessment of the target joint and enthesis. Finally, we evaluated changes in the composition of the intestinal microbiota using next-generation sequencing analysis of fecal samples. Results: The patients in the study showed a significant improvement in all clinical scores used; this improvement was also confirmed by a significant reduction in the US synovitis scores. The data on the microbiota analysis suggested that the effectiveness of tofacitinib in ameliorating PsA activity was associated with a relevant modification of some gut bacterial lineages. No cases of severe adverse effects were reported. Conclusions: Treatment with tofacitinib proved to be effective, safe and capable of varying the composition of the gut microbiota by selecting bacterial strains considered beneficial in immune modulation.

## 1. Introduction

Psoriatic arthritis (PsA) belongs to the multifaceted group of Spondyloarthritis (SpA).

It typically manifests in young individuals, peaking between the ages of 30 and 50 years, and is associated with functional impairment and a decline in health-related quality of life [1]. While peripheral arthritis is the most common clinical manifestation of PsA, axial involvement (referred to as axPsA) can be present in up to 50% of PsA cases [2]. In addition to joint inflammation, peri-articular tissues are often affected, with conditions such as enthesitis, tenosynovitis, and dactylitis [3].

Recent findings have highlighted the essential role of the gut microbiota in regulating immune balance and homeostasis in autoimmune diseases. Disruption in gut microbiota composition and diversity, known as dysbiosis, may erroneously guide the immune system toward pro-inflammatory pathways, initiating various autoimmune responses [4]. Specifically, gut dysbiosis may contribute to joint pathology through two potential mechanisms: the translocation of intestinal microbes due to increased permeability of the intestinal epithelial cell layer or exposure to microbial products [5,6,7,8]. A few studies that analyzed the composition of gut microbiota in SpA patients showed contrasting data. Costello et al. evaluated biopsy specimens from early SpA patients, finding a greater abundance of five bacterial families: *Lachnospiraceae, Ruminococcaceae, Rikenellaceae, Porphyromonadaceae,* and *Bacteroidaceae*, accompanied by a reduction in *Veillonellaceae and Prevotellaceae,* compared to healthy controls (HCs) [9]. Stebbings et al., using molecular analysis, identified a higher proportion of sulfate-reducing bacteria in AS patients than in HCs. Fecal samples and blood specimens from children with enthesitis showed a decrease in *Faecalibacterium prausnitzii* spp. and members of the *Lachnospiraceae* family, along with a significant increase in the *Bifidobacterium* genus [10]. Other studies have reported a significant increase in *Firmicutes* and a reduction in *Actinobacteria* phylum members and *Propionibacteria* spp. in the skin samples of patients with psoriasis compared to HCs [7,11,12].

In recent years, there has been growing interest in the reciprocal influence of microbiota composition and immunosuppressive drugs and how this interaction may impact clinical outcomes [5,13,14]. Immunosuppressants, such as cyclophosphamide and methotrexate, can drastically reduce gut microbiota diversity, leading to a decrease in commensal anaerobic species and an increase in potential pathogens [15,16]. This shift can damage the gut barrier, alter epithelial cell permeability, and result in bacterial translocation [17]. However, there are limited data on how biotechnological and targeted immunosuppressive drugs affect the microbiota.

Tofacitinib is the first of this new class of targeted synthetic disease-modifying anti-rheumatic drugs (tsDMARDs). It is an oral inhibitor of the enzymes JAK1, JAK2, and JAK3, which was approved in 2012 for the treatment of moderate to severe rheumatoid arthritis (RA) [18]. More recently, the efficacy and safety of tofacitinib have been demonstrated in pivotal randomized controlled trials (RCTs) and a long-term extension involving patients with psoriatic arthritis (PsA) [19,20,21,22]. A recent US real-world cohort study of PsA patients showed that tofacitinib was effective across multiple PsA domains at 6 ± 3 months of follow-up [23]. However, there is still a lack of extensive research on the drug’s effectiveness in real-world clinical practice. Very recent studies investigating the relationship between JAK inhibitors and microbiota have shown only minor effects of this kind of drug on the gut microbial taxonomic profile [24,25]; however, while one study also reported no significant impact of tofacitinib on gut microbial richness expressed by the Chao1 index in a rat model of Crohn’s disease [24], another human study highlighted the ability of JAK inhibitors to stimulate gut bacterial growth, as evidenced by an increased number of raw reads counts [25].

The aim of this study was to analyze the efficacy of tofacitinib in controlling various domains of disease activity in a case series of bio-naïve PsA patients over a 12-month follow-up period, confirmed by ultrasonographic (US) evaluation. Additionally, the study evaluated the impact of tofacitinib treatment on the composition of the gut microbiota and investigated possible correlations between the microbiota composition and immunological, clinical, and US parameters of disease activity.

## 2. Methods

### 2.1. Patients and Study Design

This was a 12-month case series report.

Seven consecutive bio-naïve patients with moderate to severe PsA disease activity (classified according to the CASPAR criteria) were enrolled.

Moderate to severe activity was defined in the presence of more than four swollen joints, and/or a Disease Activity in PSoriatic Arthritis (DAPSA) score ≥15, and/or an Ankylosing Spondylitis Disease Activity Score (ASDAS-CRP) ≥1.3, and/or a Bath Ankylosing Spondylitis Disease Activity Index (BASDAI) score ≥4. The patients were inadequate responders to csDMARDs and on their first attempt at biological therapy (bio-naïve).

At T0 (week 0/enrollment visit), T1 (12 weeks), T2 (24 weeks), and T3 (52 weeks), the following procedures were performed:

- Health and clinical history;
- Clinical examination and assessment of disease activity [DAPSA, ASDAS, BASDAI, LEI (Leeds Enthesitis Index), and PASI (Psoriasis Area Severity Index)];
- Ultrasonography (US) of target joints and enthesis, as reported in the Section 2.4.

At T0 and T3, a venous blood sample and a fresh stool sample in a tube filled with a DNA stabilization buffer were also collected for the analysis of cytokines and gut microbiota.

Tofacitinib treatment was started according to the EMA SmPC and clinical judgment, independently of the patients’ participation in the study, at the approved standard dose for PsA of 5 mg twice daily.

### 2.2. Cytokines Analysis

Serum levels of the following cytokines: IL2, IL4, IL6, IL10, IL7, IL12p70, IL17A, INFgamma, and TNFalpha were measured with Luminex assays, using commercially available kits according to the manufacturer’s instructions.

### 2.3. Next-Generation Sequencing of Bacterial 16S rRNA Gene

Microbial genomic DNA was extracted from the samples using the QIAamp Fast DNA Stool Mini Kit (Qiagen, Hilden, Germany), as described by the manufacturer. Briefly, the procedure is a spin column-based DNA purification, which uses proprietary buffers to allow the lysis of bacterial cells, the binding of nucleic acids to the silica matrix in the column, some washes to remove salts, impurities, and chemicals from the membrane, and finally the elution of purified genomic DNA. The 16S rRNA gene sequencing was carried out as described in detail in [26]. Briefly, the V3–V4 hypervariable regions of the bacterial 16S ribosomal RNA gene were amplified by PCR using barcoded universal primers. The PCR products were purified and quantified, and the equimolar ratios of the amplicons from the individual samples were pooled before multiplex paired-end sequencing (2 × 300 bp) on an Illumina MiSeq instrument (Illumina, llumina, San Diego, CA, USA). The sequences were analyzed using the 16S Metagenomics GAIA 2.0 software (Sequentia Biotech, Barcelona, Spain), in which the sequences were quality filtered based on FastQC and BBDuck, and the taxonomy was assigned using the BWA-MEM alignment against the 16S reference database available in the NCBI GenBank.

### 2.4. Ultrasonography

US was performed by an experienced rheumatologist trained in musculoskeletal US and blinded to the clinical data, using an ESAOTE (version 23.01.00) MyLab twice equipped with a multi-frequency 10–18 MHz linear transducer. All involved joints were examined in longitudinal and transverse scans according to a multiplanar scanning technique, following internationally approved guidelines. The involved joints and the following entheses were scanned: the Achilles tendon, the proximal and distal insertion of the patellar tendon, the quadriceps tendon, and the common extensor tendon. The following lesions were recorded at each site according to the Outcome Measures in Rheumatology (OMERACT) task force [26]: tendon thickness and hypo-echogenicity, bony erosions, calcifications, enthesophytes, and a power Doppler (PD) signal within 2 mm of the tendon insertion. The presence of synovial hypertrophy and a PD signal was registered following the OMERACT definitions, using both grayscale (GS) and PD modalities and were scored according to a semiquantitative scale (0 = absent, 1 = mild, 2 = moderate, and 3 = severe). The PD settings were the following: frequency 8.3–10 MHz, pulse repetition frequency 600 Hz, and gain-adjusted just below the level that caused the appearance of noise artifacts and a low wall filter.

The Global EULAR-OMERACT Synovitis Score (GLOESS) was then calculated as the sum of each PDUS composite score for all joints examined [27].

## 3. Statistical Analysis

The statistical analysis was performed using R version 4.3.1 (16 June 2023 ucrt)—“Beagle Scouts” Copyright © 2023, the R foundation for Statistical Computing. The continuous data are described as median (25th–75th percentile) or mean and standard deviation (SD), while the categorical variables are described as percentages (%). The D’Agostino–Pearson test was used to test the normality of the data. Fisher’s exact test was used for analysis of the categorical variables, while a repeated measures ANOVA test (with a Holmes post hoc test) was used to compare the DAPSA, ASDAS, BASDAI, LEI, PASI, tender joints, and swollen joints variables. The Kruskal–Wallis test was used for the time course of the cytokines. Correlation analyses were performed using the Spearman rank correlation and R’s corrplot library showing only statistically significant correlations, positive or negative, diversified by color gradient. The results were considered significant when *p* <0.05.

The differential analysis of the gut microbiota composition was performed using DESeq2 statistics (Version 1.44.0). The results were considered significant when the FDR-adjusted *p*-value was <0.05.

## 4. Results

A total of seven PsA patients were enrolled and completed 52 weeks of follow-up (T3) (Table 1).

Regarding the arthritis phenotype, four patients had peripheral joint involvement only, one had axial involvement only, and two had a mixed form. Enthesitis was present in four patients, while four had psoriasis (PsO), and three had a past or family history of PsO. None of the patients had ocular or intestinal involvement.

At baseline, four patients had moderate activity, while three had high or very high activity.

At the 52-week mark (T3), the DAPSA and PASI scores significantly reduced from 25.8 to 13.97 (*p =* 0.001) and from 3.86 to 1 (*p =* 0.03), respectively. A significant decrease in the mean number of tender joints from 7 to 3.5 (*p =* 0.028) was also observed (Table 2).

**Figure 1:**
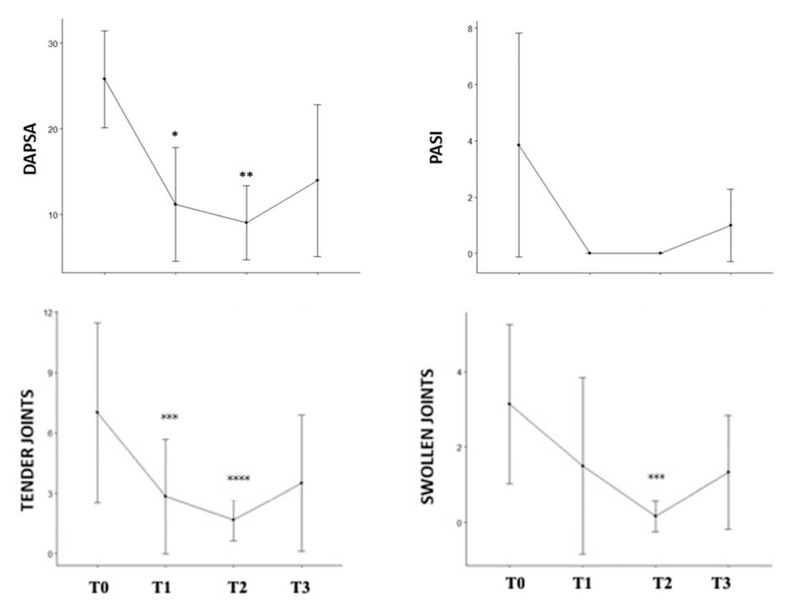
DAPSA, PASI scores, and number of tender and swollen joints throughout the follow-up of the psoriatic arthritis patients. DAPSA = Disease activity in Psoriatic Arthritis; PASI = Psoriasis Area Severity Index * *p* = 0.03; ** *p* = 0.008; *** *p* = 0.01; **** p = 0.04.

Four patients achieved low disease activity, while the remaining three achieved moderate disease activity. Notably, the most substantial decline occurred at 12 weeks (T1) and continued steadily at 24 weeks (T2) (Figure 1). Between 24 weeks (T2) and 52 weeks (T3), there was a mild worsening of articular symptoms in two patients, causing a slight increase in the disease activity parameters, although the overall reduction in disease activity at 52 weeks remained significant (*p =* 0.001). There was also a significant noticeable decrease in the number of swollen joints from T0 to T2, which was statistically significant (Figure 1), and a resolution of enthesitis in three out of four patients. Regarding the serologic parameters, from T0 to T3, there was a tendency towards reduced CRP and ESR levels, even though the change was not statistically significant (Supplementary Materials).

These results were supported by the significant reduction in the GLOESS score, which dropped from 7 to 3 after 12 weeks, with further improvement at 52 weeks (Table 2). Finally, no significant differences in serum cytokine levels were observed before and after the tofacitinib treatment (Supplementary Materials).

During the follow-up, neither dropouts nor serious adverse events (SAEs) were registered.

**Figure 2:**
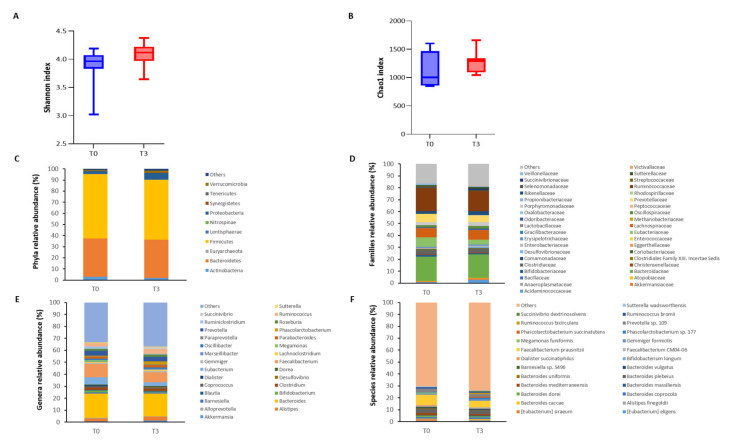
Effect of tofacitinib on gut microbiota alpha diversity and taxonomic composition. Boxplots representing species-level diversity expressed by Shannon Index (**A**) and richness expressed by Chao1 index (**B**) before and after treatment. Stacked barplots showing the mean relative abundance of gut bacteria at the phylum (**C**), family (**D**), genus (**E**), and species (**F**) level, before and after treatment.

The gut microbiota of the seven patients enrolled in the study was analyzed at baseline (T0) and after 52 weeks (T3) of treatment by 16S rRNA gene sequencing, yielding an average of 127,304.9 quality-filtered read pairs per sample. Though not affecting the alpha-diversity (Figure 2A) and richness (Figure 2B) indices, the tofacitinib treatment produced remarkable modifications in the gut microbiota composition (Figure 2C–F), as described in Table 3.

The bacteria identified at each taxonomic level were then correlated with the immunological and clinical parameters of the patients, both at T0 and at T3. The Pearson’s correlation analyses revealed that the associations were quite different before and after the tofacitinib treatment. Remarkably, a number of significant sporadic correlations were observed at baseline. Among them, at the phylum level, *Tenericutes* were found to positively correlate with TNF-alpha and CRP, *Bacteroidetes* were positively associated with the DAPSA, while *Verrucomicrobia* was negatively associated with the ESR and with the PASI, and *Firmicutes* showed an inverse correlation with the DAPSA. Among the strongest associations observed at the family level, *Selenomonadaceae* were positively correlated with the DAPSA and negatively correlated with IL-22, *Gracilibacteraceae* were inversely correlated with the ESR, DAPSA, and PASI, *Eggerthellaceae* showed a negative association with the DAPSA, *Prevotellaceae* and *Sutterellaceae* were directly associated with the DAPSA, and *Akkermansiaceae* and *Rhodospirillaceae* were negatively correlated with the PASI.

At the genus level, strong positive correlations between *Bilophila* and IL-10 and between *Coprobacter* and IL-6 and negative correlations between *Ihubacter* and IL-6, *Marseilla* and IL-22, and *Prevotella* and IL22 were observed.

**Figure 3:**
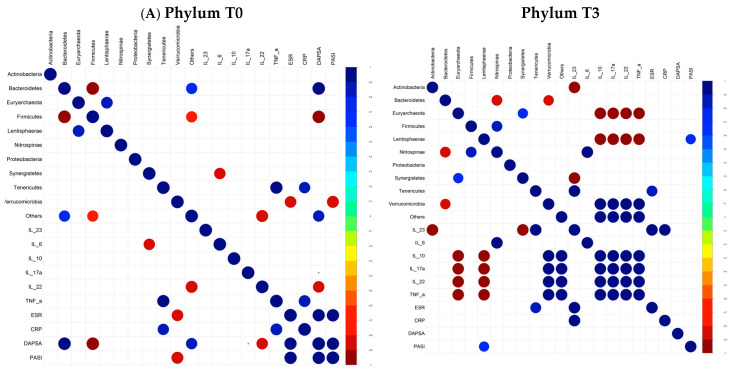

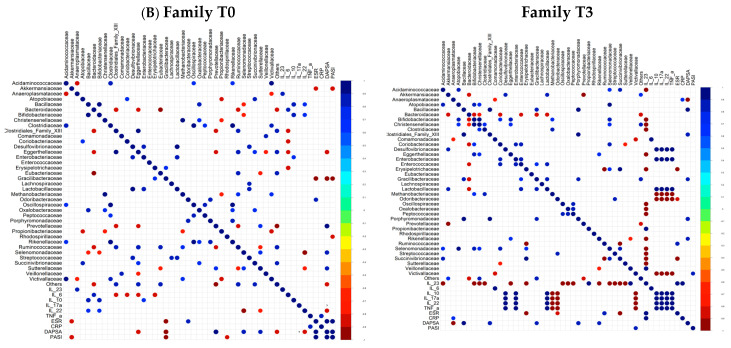
Association of gut microbiota profiles with clinical and serologic parameters in psoriatic arthritis patients at the phylum (**A**) and family (**B**) levels at T0 and T3.

On the contrary, after the treatment, certain bacteria were shown to be significantly correlated with groups of cytokines/parameters; for instance, at the phylum level, *Euryarcheota* and *Lentisphaerae* were negatively associated, while *Verrucomicrobia* were positively associated with IL-10, IL-22, IL-17a, and TNF-a (Figure 3A); in addition, *Lentisphaerae* were positively associated with the PASI. At the family level, the same abovementioned cytokines were positively associated with *Desulfovibrionaceae*, *Enterobacteriaceae,* and *Lactobacillaceae* and negatively associated with *Methanobacteriaceae*, *Odoribacteriaceae,* and *Victivallaceae*; moreover, *Anaeroplasmataceae* showed a negative association, while *Bacillaceae*, *Clostridiales Family XIII*, *Gracilibacteraceae*, *Porphyromonadaceae*, and *Rikenellaceae* showed a positive association with the DAPSA; *Victivallaceae* was positively correlated with the PASI (Figure 3B).

Again, at the genus level the same group of cytokines was positively correlated with *Bilophila*, *Butyrivibrio*, *Coprobacter*, *Coprococcus*, *Eisenbergiella*, *Enterococcus*, *Eubacterium*, *Lactobacillus*, *Parabacteroides*, and *Robinsoniella* and negatively correlated with *Anaeromassilibacillus*, *Catenibacterium*, *Dorea*, *Enterohabdus*, *Flintibacter*, *Gemmiger*, *Harryflintia*, *Mediterranea*, *Methanobrevibacter*, *Parasutterella*, and *Victivallis*.

## 5. Discussion

Tofacitinib proved effective in both articular and cutaneous manifestations, significantly reducing the DAPSA and PASI scores, as well as the number of tender joints. Improvements in arthritis were rapid, with the most notable decline occurring between T0 and T1, during which 57% of the patients achieved a low disease activity state. While the number of swollen joints also decreased, this reduction was not statistically significant, likely due to the small sample size.

These clinical improvements were corroborated by the ultrasonographic findings, which showed a significant reduction in the GLOESS score. On the other hand, we did not observe significant modifications in the serum cytokines levels, probably due to the limited number of enrolled subjects.

Importantly, tofacitinib was well tolerated, with no reports of SAEs or therapy discontinuation.

Several RCTs and observational studies have demonstrated that tofacitinib induces rapid and sustained improvements in the skin, joints, spine, entheses, and overall quality of life of PsA patients [20,21,22,23,28].

Current EULAR guidelines recommend the use of JAK-i after the failure of at least one bDMARD, considering factors such as the efficacy–safety balance, cost, and long-term experience [29]. Conversely, the Group for Research and Assessment of Psoriasis and Psoriatic Arthritis (GRAPPA) task force recommends bDMARDs and JAK-i equally, in particular for patients with predominant joint or axial disease [30].

To the best of our knowledge, this is the first study assessing through US the efficacy of tofacitinib in reducing joint inflammation in patients with PsA. Previous observational studies have primarily focused on rheumatoid arthritis (RA) [31,32]. A recent multicenter Italian prospective study reported a significant reduction in US joint and tendon scores after just 2 weeks of tofacitinib treatment in RA patients, with effects lasting up to 24 weeks [32].

Given the established efficacy and safety of tofacitinib in inflammatory bowel disease [33] and the well-documented connection between gut bacteria and rheumatic diseases, we also explored tofacitinib’s impact on gut microbiota composition, along with potential correlations between microbiota changes and clinical disease activity. Notably, we observed a significant decrease in the phylum *Actinobacteria*, which has been reported as elevated in both PsA and RA patients [34,35,36]. Moreover, the genus *Megamonas* (and its related species *Megamonas rupellensis* and *Megamonas funiformis*) also decreased significantly after tofacitinib treatment. This bacterium has been overrepresented in several autoimmune and rheumatic diseases, which are somehow related to PsA, including psoriasis [37], inactive systemic lupus erythematosus (iSLE) [38], primary Sjögren’s Syndrome (pSS) [39], and rheumatoid arthritis [32]. *Megamonas* abundance has also been associated with elevated serum levels of IL-10, IL-2, IL-4, TNF-a, and IFN-g, as well as with the Th1 and Th2 CD4+ T-cell subpopulation in RA patients [36], and with markers of disease either in iSLE [38] and in pSS [39].

Conversely, different butyrate-producing bacterial species (i.e., *Coprococcus comes*, *Ruminococcus bicirculans*, and *Butyricimonas* sp. AT11) became enriched after the tofacitinib therapy. Butyrate is an anti-inflammatory short-chain fatty acid that has been detected in lower levels in psoriatic patients progressing to PsA [40]. This suggests a possible role for this compound in the pathogenesis of the disease. Interestingly, *Coprococcus comes* was virtually absent in the PsA patients at baseline but increased significantly after treatment. A previous study noted the depletion of this bacterium in patients with chronic widespread musculoskeletal pain, which is a common symptom of fibromyalgia and other rheumatic diseases [41]. Consistently, the *Coprococcus* genus has been reported as decreased in PsA [42] and RA patients [36] compared to healthy controls.

The impact of tofacitinib on mucosal immunity and gut microbiota has been previously evaluated in a collagen-induced arthritis model in DBA1/J mice. While there were some differences in microbiota composition compared to our PsA findings—likely due to the differences in disease models—that study reported a decrease in *Proteobacteria* and an increase in certain *Actinobacteria* members, such as *Coriobacteriales* and *Enterohabdus*. It also noted a reduction in potentially pathogenic *Chlamydiae* [43].

When the gut microbial levels were correlated with clinical inflammatory markers, a number of taxa showed significant positive or negative associations with groups of inflammatory cytokines, especially following tofacitinib treatment. At the baseline, some bacteria previously implicated in inflammatory conditions were positively associated with the DAPSA score. These included *Selenomonadaceae*, which were increased in the RA and osteoarthritis patients [44], *Prevotellaceae*, again found enriched in the RA patients [45], and *Eggerthellaceae*, whose increase was described in the patients with psoriasis [46].

As far as we know, this is the first study evaluating the effect of tofacitinib on gut microbiota in PsA patients.

The main limit of this case series that needs to be considered is the small number of enrolled subjects and the monocentric nature of the study, which do not allow generalization of the results obtained.

On the other hand, the comprehensive clinical evaluation of various PsA domains, the incorporation of musculoskeletal US assessments, and the detailed analysis of gut microbiota before and after tofacitinib treatment represent a strength of the study.

## 6. Conclusions

In this case series of PsA patients, tofacitinib demonstrated a significant reduction in disease activity across both the articular and cutaneous domains. This clinical improvement was further supported by a significant decrease in the ultrasonographic synovitis score. Additionally, the tofacitinib treatment was associated with notable changes in gut bacterial lineages, including an increase in butyrate-producing bacterial species.

Taken together, these results support the effectiveness of tofacitinib in improving disease activity and show for the first time its ability to modify some gut bacterial lineages in a small sample of patients with PsA.

## Figures and Tables

**Table 1:** Baseline characteristics of enrolled psoriatic arthritis patients.

Patients’ Characteristics	
Total (n)	7
Age, median (IQR) years	52.00 (33.00–56.00)
Gender, Female (n/tot)	57.14% (4/7)
Comorbidities, yes (n/tot)	57.14% (4/7)
BMI, median (IQR)	25.7 (23–26.6)
Smoking habits, yes (n/tot)	28.57% (2/7)
Disease duration, median (IQR) years	10.34 (0.4–13)
ESR, median (IQR) mm/hr	8.00 (2.00–68.00)
CRP, median (IQR) mg/dL	0.40 (0.10–4.00)
Swollen joints, mean (SD)	3.1 (2.1)
Tender joints, mean (SD)	7 (4.4)
LEI, mean (SD)	1 (0.9)
PASI, mean (SD)	3.86 (3.9)
DAPSA, mean (SD)BASDAI, mean (SD)ASDAS, mean (SD)	25.8 (5.6)7.00 (0.14)4.35 (0.1)
Monotherapy (n/tot)	42.85% (3/7)
GLOESS score, mean (SD)	7.00 (7.7)

BMI = Body mass index; LEI = Leeds Enthesitis Index; PASI = Psoriasis Area Severity Index; DAPSA = Disease Activity in PSoriatic Arthritis; BASDAI = Bath Ankylosing Spondylitis Disease Activity Index; ASDAS = Ankylosing Spondylitis Disease Activity Score; GLOESS = Global EULAR and OMERACT Synovitis Score; CRP = C-reactive protein; ESR = Erythrocyte sedimentation rate.

**Table 2:** Clinical and ultrasonography scores throughout the follow-up of the psoriatic arthritis patients.

	T0	T1	T2	T3	*p*-Value *
DAPSA, mean (SD)	25.80 (5.65)	9.40(5.64)	8.40 (4.51)	13.97 (8.88)	0.001
ASDAS, mean (SD)	4.35 (1.34)	2.40 (NA)	4.20 (NA)	2.50 (NA)	-
BASDAI, mean (SD)	7.00 (0.14)	1.60 (NA)	4.20 (NA)	3.10 (NA)	-
LEI, mean (SD)	1 (0.90)	0.50 (1.00)	0.40 (0.89)	0.9 (0.70)	0.482
PASI, mean (SD)	3.86 (3.98)	0.00 (0.00)	0.00 (0.00)	1.00 (1.29)	0.030
Tender joints, mean (SD)	7.00 (4.47)	1.40 (2.61)	0.20 (0.45)	3.50 (3.39)	0.028
Swollen joints, mean (SD)	3.14 (2.12)	1.40 (2.61)	0.20 (0.45)	1.33 (1.51)	0.083
GLOESS, mean (SD)	7.00 (7.72)	3.00 (3.37)	1.57 (1.40)	2.00 (2.77)	0.122

LEI = Leeds Enthesitis Index; PASI = Psoriasis Area Severity Index; DAPSA = Disease Activity in PSoriatic Arthritis; BASDAI = Bath Ankylosing Spondylitis Disease Activity Index; ASDAS = Ankylosing Spondylitis Disease Activity Score; GLOESS = Global EULAR and OMERACT Synovitis Score * Repeated measures ANOVA test (with Holmes post hoc test).

**Table 3:** Significant changes in microbiota composition between T0 and T3.

	Relative Abundance at T0 (%)	Relative Abundance at T3 (%)
Increased at T3		
*Lentisphaerae*	0.073	0.410
*Proteobacteria*	2.160	6.018
*Oxalobacteraceae*	0.009	0.048
*Desulfovibrionaceae*	0.258	0.730
*Phascolarctobacterium*	0.398	2.366
*Angelakisella*	0.015	0.049
*Bilophila*	0.067	0.189
*Anaeromassilibacillus*	0.014	0.044
*Coprococcus comes*	0.000	0.059
*Alistipes* sp. NML05A004	0.000	0.045
*Sutterella massiliensis*	0.022	0.290
*Ruminococcus bicirculans*	0.242	0.997
*Butyricimonas* sp. AT11	0.008	0.042
*Bilophila wadsworthia*	0.034	0.121
*Clostridium leptum*	0.012	0.047
*Parabacteroides distasonis*	0.153	0.497
Decreased at T3		
*Actinobacteria*	2.988	1.879
*Selenomonadaceae*	1.344	0.070
*Coriobacteraceae*	0.686	0.484
*Megamonas*	1.304	0.001
*Adelcreutzia*	0.051	0.012
*Enterohabdus*	0.065	0.025
*Ruminoclostridium*	1.708	0.550
*Collinsella*	0.580	0.412
*Veillonella*	0.127	0.069
*Megamonas rupellensis*	0.090	0.000
*Megamonas funiformis*	0.997	0.001
*Bacteroides galacturonicus*	0.119	8.571 × 10^−5^
*Tyzzerella nexilis*	0.049	0.001
*Dialister* sp. S7D	0.096	0.027
*Eubacterium hallii*	0.185	0.078
*Parasutterella excrementihominis*	0.072	0.036

## Data Availability

The data that support the findings of this study are available from the authors’ databases upon reasonable request.

## Supplementary Materials

The following supporting information can be downloaded at: https://www.mdpi.com/article/10.3390/microorganisms12122387/s1, Table S1: Cytokines and inflammatory markers throughout the follow-up in the psoriatic arthritis patients.

## References

[1] Hernández-Rodríguez J.C., Infante-Cano M., García-Muñoz C., Matias-Soto J., Martinez-Calderon J. (2024). Psoriatic arthritis with psychological comorbidities: An overview of systematic reviews on incidence, prevalence, and geographic disparities. Rheumatol. Int..

[2] Chandran V., Barrett J., Schentag C.T., Farewell V.T., Gladman D.D. (2009). Axial psoriatic arthritis: Update on a longterm prospective study. J. Rheumatol..

[3] Furtunescu A.R., Georgescu S.R., Tampa M., Matei C. (2024). Inhibition of the JAK-STAT Pathway in the Treatment of Psoriasis: A Review of the Literature. Int. J. Mol. Sci..

[4] Shaheen W.A., Quraishi M.N., Iqbal T.H. (2022). Gut microbiome and autoimmune disorders. Clin. Exp. Immunol..

[5] Zhao T., Wei Y., Zhu Y., Xie Z., Hai Q., Li Z., Qin D. (2022). Gut microbiota and rheumatoid arthritis: From pathogenesis to novel therapeutic opportunities. Front. Immunol..

[6] Thye A.Y., Bah Y.R., Law J.W., Tan L.T., He Y.W., Wong S.H., Thurairajasingam S., Chan K.G., Lee L.H., Letchumanan V. (2022). Gut-Skin Axis: Unravelling the Connection between the Gut Microbiome and Psoriasis. Biomedicines.

[7] Wang Y., Wei J., Zhang W., Doherty M., Zhang Y., Xie H., Li W., Wang N., Lei G., Zeng C. (2022). Gut dysbiosis in rheumatic diseases: A systematic review and meta-analysis of 92 observational studies. EBioMedicine.

[8] Jiao Y., Wu L., Huntington N.D., Zhang X. (2020). Crosstalk Between Gut Microbiota and Innate Immunity and Its Implication in Autoimmune Diseases. Front. Immunol..

[9] Costello M.E., Ciccia F., Willner D., Warrington N., Robinson P.C., Gardiner B., Marshall M., Kenna T.J., Triolo G., Brown M.A. (2015). Brief Report: Intestinal Dysbiosis in Ankylosing Spondylitis. Arthritis Rheumatol..

[10] Stebbings S., Munro K., Simon M.A., Tannock G., Highton J., Harmsen H., Welling G., Seksik P., Dore J., Grame G. (2002). Comparison of the faecal microflora of patients with ankylosing spondylitis and controls using molecular methods of analysis. Rheumatology.

[11] Salvadori M., Rosso G. (2024). Update on the reciprocal interference between immunosuppressive therapy and gut microbiota after kidney transplantation. World J. Transplant..

[12] Picchianti-Diamanti A., Panebianco C., Salemi S., Sorgi M.L., Di Rosa R., Tropea A., Sgrulletti M., Salerno G., Terracciano F., D’Amelio R. (2018). Analysis of Gut Microbiota in Rheumatoid Arthritis Patients: Disease-Related Dysbiosis and Modifications Induced by Etanercept. Int. J. Mol. Sci..

[13] Panebianco C., Andriulli A., Pazienza V. (2018). Pharmacomicrobiomics: Exploiting the drug-microbiota interactions in anticancer therapies. Microbiome.

[14] Bhat M., Pasini E., Copeland J., Angeli M., Husain S., Kumar D., Renner E., Teterina A., Allard J., Guttman D.S. (2017). Impact of Immunosuppression on the Metagenomic Composition of the Intestinal Microbiome: A Systems Biology Approach to Post-Transplant Diabetes. Sci. Rep..

[15] Olejniczak-Staruch I., Ciążyńska M., Sobolewska-Sztychny D., Narbutt J., Skibińska M., Lesiak A. (2021). Alterations of the Skin and Gut Microbiome in Psoriasis and Psoriatic Arthritis. Int. J. Mol. Sci..

[16] Thio H.B. (2018). The Microbiome in Psoriasis and Psoriatic Arthritis: The Skin Perspective. J. Rheumatol. Suppl..

[17] Harrington R., Al Nokhatha S.A., Conway R. (2020). JAK Inhibitors in Rheumatoid Arthritis: An Evidence-Based Review on the Emerging Clinical Data. J. Inflamm. Res..

[18] Ighani A., Georgakopoulos J.R., Yeung J. (2020). Tofacitinib for the treatment of psoriasis and psoriatic arthritis. G. Ital. Dermatol. Venereol..

[19] Kerschbaumer A., Smolen J.S., Ferreira R.J.O., Bertheussen H., Baraliakos X., Aletaha D., McGonagle D.G., van der Heijde D., McInnes I.B., Esbensen B.A. (2024). Efficacy and safety of pharmacological treatment of psoriatic arthritis: A systematic literature research informing the 2023 update of the EULAR recommendations for the management of psoriatic arthritis. Ann. Rheum. Dis..

[20] Mease P., Hall S., FitzGerald O., Van der Heijde D., Merola J.F., Avila-Zapata F., Cieślak D., Graham D., Wang C., Menon S. (2017). Tofacitinib or adalimumab versus placebo for psoriatic arthritis. N. Engl. J. Med..

[21] Gladman D., Rigby W., Azevedo V.F., Behrens F., Blanco R., Kaszuba A., Kudlacz E., Wang C., Menon S., Hendrikx T. (2017). Tofacitinib for psoriatic arthritis in patients with an inadequate response to TNF inhibitors. N. Engl. J. Med..

[22] Nash P., Mease P.J., Wu J., Coates L.C., Behrens F., Gladman D.D., Kivitz A.J., Wei J.C., Shirinsky I., Menon S. (2020). Tofacitinib as monotherapy following methotrexate withdrawal in patients with psoriatic arthritis previously treated with open-label tofacitinib plus methotrexate: A randomised, placebo-controlled sub-study of OPAL Balance. Lancet Rheumatol..

[23] Mease P.J., Young P., Fallon L., Mundayat R., Dina O., Blachley T., Middaugh N., Ogdie A. (2024). Effectiveness of Tofacitinib in Patients Initiating Therapy for Psoriatic Arthritis: Results from the CorEvitas Psoriatic Arthritis/Spondyloarthritis Registry. Rheumatol. Ther..

[24] Blondeaux A., Valibouze C., Speca S., Rousseaux C., Dubuquoy C., Blanquart H., Zerbib P., Desreumaux P., Foligné B., Titécat M. (2024). Changes in HLA-B27 Transgenic Rat Fecal Microbiota Following Tofacitinib Treatment and Ileocecal Resection Surgery: Implications for Crohn’s Disease Management. Int. J. Mol. Sci..

[25] Favaron A., Abdalla Y., McCoubrey L.E., Nandiraju L.P., Shorthouse D., Gaisford S., Basit A.W., Orlu M. (2024). Exploring the interactions of JAK inhibitor and S1P receptor modulator drugs with the human gut microbiome: Implications for colonic drug delivery and inflammatory bowel disease. Eur. J. Pharm. Sci..

[26] Bruyn G.A., Iagnocco A., Naredo E., Balint P.V., Gutierrez M., Hammer H.B., Collado P., Filippou G., Schmidt W.A., Jousse-Joulin S. (2019). OMERACT Ultrasound Working Group. OMERACT Definitions for Ultrasonographic Pathologies and Elementary Lesions of Rheumatic Disorders 15 Years On. J. Rheumatol..

[27] D’Agostino M.A., Schett G., López-Rdz A., Šenolt L., Fazekas K., Burgos-Vargas R., Maldonado-Cocco J., Naredo E., Carron P., Duggan A.M. (2022). Response to secukinumab on synovitis using Power Doppler ultrasound in psoriatic arthritis: 12-week results from a phase III study, ULTIMATE. Rheumatology.

[28] Dai Q., Zhang Y., Liu Q., Zhang C. (2024). Efficacy and safety of tofacitinib for chronic plaque psoriasis and psoriatic arthritis: A systematic review and meta-analysis of randomized controlled trials. Clin. Rheumatol..

[29] Gossec L., Kerschbaumer A., Ferreira R.J.O., Aletaha D., Baraliakos X., Bertheussen H., Boehncke W.H., Esbensen B.A., McInnes I.B., McGonagle D. (2024). EULAR recommendations for the management of psoriatic arthritis with pharmacological therapies: 2023 update. Ann. Rheum. Dis..

[30] Coates L.C., Soriano E.R., Corp N., Bertheussen H., Callis Duffin K., Campanholo C.B., Chau J., Eder L., Fernández-Ávila D.G., FitzGerald O. (2022). GRAPPA Treatment Recommendations domain subcommittees. Group for Research and Assessment of Psoriasis and Psoriatic Arthritis (GRAPPA): Updated treatment recommendations for psoriatic arthritis 2021. Nat. Rev. Rheumatol..

[31] Ceccarelli F., Spinelli F.R., Garufi C., Mancuso S., Alessandri C., Di Franco M., Orefice V., Pacucci V.A., Pirone C., Priori R. (2022). The role of musculoskeletal ultrasound in predicting the response to JAK inhibitors: Results from a monocentric cohort. Clin. Exp. Rheumatol..

[32] Germanò G., Macchioni P., Maranini B., Ciancio G., Bonazza S., Govoni M., Salvarani C. (2022). Ultrasound response to tofacitinib in patients with rheumatoid arthritis: Data from a multicenter 24 weeks prospective study. Front. Med..

[33] Mpakogiannis K., Fousekis F.S., Christodoulou D.K., Katsanos K.H., Narula N. (2023). The current role of Tofacitinib in acute severe ulcerative colitis in adult patients: A systematic review. Dig. Liver Dis..

[34] Lin C.Y., Hsu C.Y., He H.R., Chiang W.Y., Lin S.H., Huang Y.L., Kuo Y.H., Su Y.J. (2022). Gut microbiota differences between psoriatic arthritis and other undifferentiated arthritis: A pilot study. Medicine.

[35] Chen J., Wright K., Davis J.M., Jeraldo P., Marietta E.V., Murray J., Nelson H., Matteson E.L., Taneja V. (2016). An expansion of rare lineage intestinal microbes characterizes rheumatoid arthritis. Genome Med..

[36] Wang Q., Zhang S.X., Chang M.J., Qiao J., Wang C.H., Li X.F., Yu Q., He P.F. (2022). Characteristics of the Gut Microbiome and Its Relationship with Peripheral CD4+ T Cell Subpopulations and Cytokines in Rheumatoid Arthritis. Front. Microbiol..

[37] Zhang X., Shi L., Sun T., Guo K., Geng S. (2021). Dysbiosis of gut microbiota and its correlation with dysregulation of cytokines in psoriasis patients. BMC Microbiol..

[38] Balmant B.D., Fonseca D.C., Prudêncio A.P.A., Rocha I.M., Callado L., Alves J.T.M., Torrinhas R.S.M.M., Borba E.F., Waitzberg D.L. (2023). Megamonas funiformis, Plasma Zonulin, and Sodium Intake Affect C3 Complement Levels in Inactive Systemic Lupus Erythematosus. Nutrients.

[39] Li Y., Li Z., Sun W., Wang M., Li M. (2022). Characteristics of gut microbiota in patients with primary Sjögren’s syndrome in Northern China. PLoS ONE.

[40] Paine A., Brookes P.S., Bhattacharya S., Li D., De La Luz Garcia-Hernandez M., Tausk F., Ritchlin C. (2023). Dysregulation of Bile Acids, Lipids, and Nucleotides in Psoriatic Arthritis Revealed by Unbiased Profiling of Serum Metabolites. Arthritis Rheumatol..

[41] Freidin M.B., Stalteri M.A., Wells P.M., Lachance G., Baleanu A.F., Bowyer R.C.E., Kurilshikov A., Zhernakova A., Steves C.J., Williams F.M.K. (2021). An association between chronic widespread pain and the gut microbiome. Rheumatology.

[42] Scher J.U., Ubeda C., Artacho A., Attur M., Isaac S., Reddy S.M., Marmon S., Neimann A., Brusca S., Patel T. (2015). Decreased bacterial diversity characterizes the altered gut microbiota in patients with psoriatic arthritis, resembling dysbiosis in inflammatory bowel disease. Arthritis Rheumatol..

[43] Hablot J., Ferhat M., Lavelle A., Salem F., Taieb M., Medvedovic J., Kindt N., Reboul P., Cailotto F., Jouzeau J.Y. (2020). Tofacitinib treatment alters mucosal immunity and gut microbiota during experimental arthritis. Clin. Transl. Med..

[44] Lee J.Y., Mannaa M., Kim Y., Kim J., Kim G.T., Seo Y.S. (2019). Comparative Analysis of Fecal Microbiota Composition Between Rheumatoid Arthritis and Osteoarthritis Patients. Genes.

[45] Alpizar-Rodriguez D., Lesker T.R., Lesker T.R., Gronow A., Gilbert B., Raemy E., Lamacchia C., Gabay C., Finckh A., Strowig T. (2019). Prevotella copri in individuals at risk for rheumatoid arthritis. Ann. Rheum. Dis..

[46] Hidalgo-Cantabrana C., Gómez J., Gómez J., Delgado S., Requena-López S., Queiro-Silva R., Margolles A., Coto E., Sánchez B., Coto-Segura P. (2019). Gut microbiota dysbiosis in a cohort of patients with psoriasis. Br. J. Dermatol..

