# Supplementary material for: A Case Series Report on the Effect of Tofacitinib on Joint Inflammation and Gut Microbiota Composition in Psoriatic Arthritis Patients Naive to Biologic Agents
Source: Microorganisms. 2024 Nov 21;12(12):2387. doi: 10.3390/microorganisms12122387 (PMC11676042; doi:10.3390/microorganisms12122387)
Supplement: Supplementary file 1 [file microorganisms-12-02387-s001.zip › microorganisms-3321190-supplementary.pdf]

Supplementary Materials

Table S1. Cytokines and inflammatory markers throughout the follow-up in the psoriatic arthritis patients.

	**T0**	**T3**	***p*-Value**
IL-23 ng/mL, median(IQR)	0.85 [0.83, 0.87]	0.84 [0.84, 1.05]	0.440
IL-6 ng/mL, median(IQR)	2.50 [0.47, 44.43]	2.50 [2.50, 91.40]	0.680
IL-10 ng/mL, median(IQR)	2.74 [2.44, 9.59]	2.62 [2.60, 12.40]	0.768
IL-17A ng/mL, median(IQR)	12.00 [0.58, 12.00]	3.74 [1.30, 12.00]	0.153
IL- 22 ng/mL, median(IQR)	0.15 [0.14, 0.17]	0.16 [0.14, 0.17]	0.895
TNF-α ng/mL, median(IQR)	7.91 [7.30, 10.45]	9.92 [8.20, 16.60]	0.413
ESR, mm/h, median(IQR)	8.00 [2.00, 68.00]	7.00 [2.00, 38.00]	0.699
RCP, mg/dL, median(IQR)	0.40 [0.10, 4.00]	0.08 [0.00, 1.14]	0.074
